# Pleural empyema caused by dropped gallstones after laparoscopic cholecystectomy for acute cholecystitis: a case report

**DOI:** 10.1186/s40792-022-01419-4

**Published:** 2022-04-07

**Authors:** Aya Tokuda, Hiromitsu Maehira, Hiroya Iida, Haruki Mori, Nobuhito Nitta, Takeru Maekawa, Katsushi Takebayashi, Sachiko Kaida, Toru Miyake, Ryo Kuroda, Haruka Yamamoto, Masaji Tani

**Affiliations:** 1 Department of Surgery, Shiga University of Medical Science, Seta-tsukinowacho, Otsu, Shiga 520-2192 Japan; 2 Division of Respiratory Medicine, Department of Internal Medicine, Shiga University of Medical Science, Otsu, Shiga Japan

**Keywords:** Pleural empyema, Dropped gallstone, Acute cholecystitis, Laparoscopic cholecystectomy

## Abstract

**Background:**

Dropped gallstones during laparoscopic cholecystectomy (LC) sometimes induce postoperative infectious complications. However, pleural empyema rarely occurs as a complication of LC.

**Case presentation:**

We present the case of a 66-year-old woman with right pleural empyema. She previously underwent LC for acute gangrenous cholecystitis 11 months ago. The operative report revealed iatrogenic gallbladder perforation and stone spillage. The bacterial culture of the gallbladder bile was positive for *Escherichia coli*. Chest and abdominal computed tomography revealed right pleural effusion, perihepatic fluid collection, and multiple small radiopaque density masses. Although ultrasound-guided transthoracic drainage was performed, the drainage was incomplete, and systemic inflammatory reaction persisted. Consequently, thoracotomy and laparotomy with gallstone retrieval were performed, and the patient recovered completely. The patient has remained well without complications after 14 months of follow-up.

**Conclusions:**

We report a rare case of pleural empyema caused by dropped gallstones after LC. This case emphasized the importance of completely retrieving the dropped gallstones to prevent late infectious complications after LC.

## Background

Laparoscopic cholecystectomy (LC) is the gold standard for cholelithiasis owing to shorter postoperative stay and fewer complications [1–3]. Furthermore, LC is the first treatment option for acute cholecystitis [4]. However, intraoperative gallbladder perforation and gallstone spillage sometimes occur during LC [5, 6].

Dropped gallstones during LC sometimes induce postoperative infectious complications, such as abdominal abscesses [7]. However, pleural empyema rarely occurs as a complication of LC. This study aimed to highlight the potential risk of dropped gallstones in the patients’ postoperative course and to provide a review of literature on pleural empyema caused by dropped gallstones during LC.

## Case presentation

**Fig. 1:**
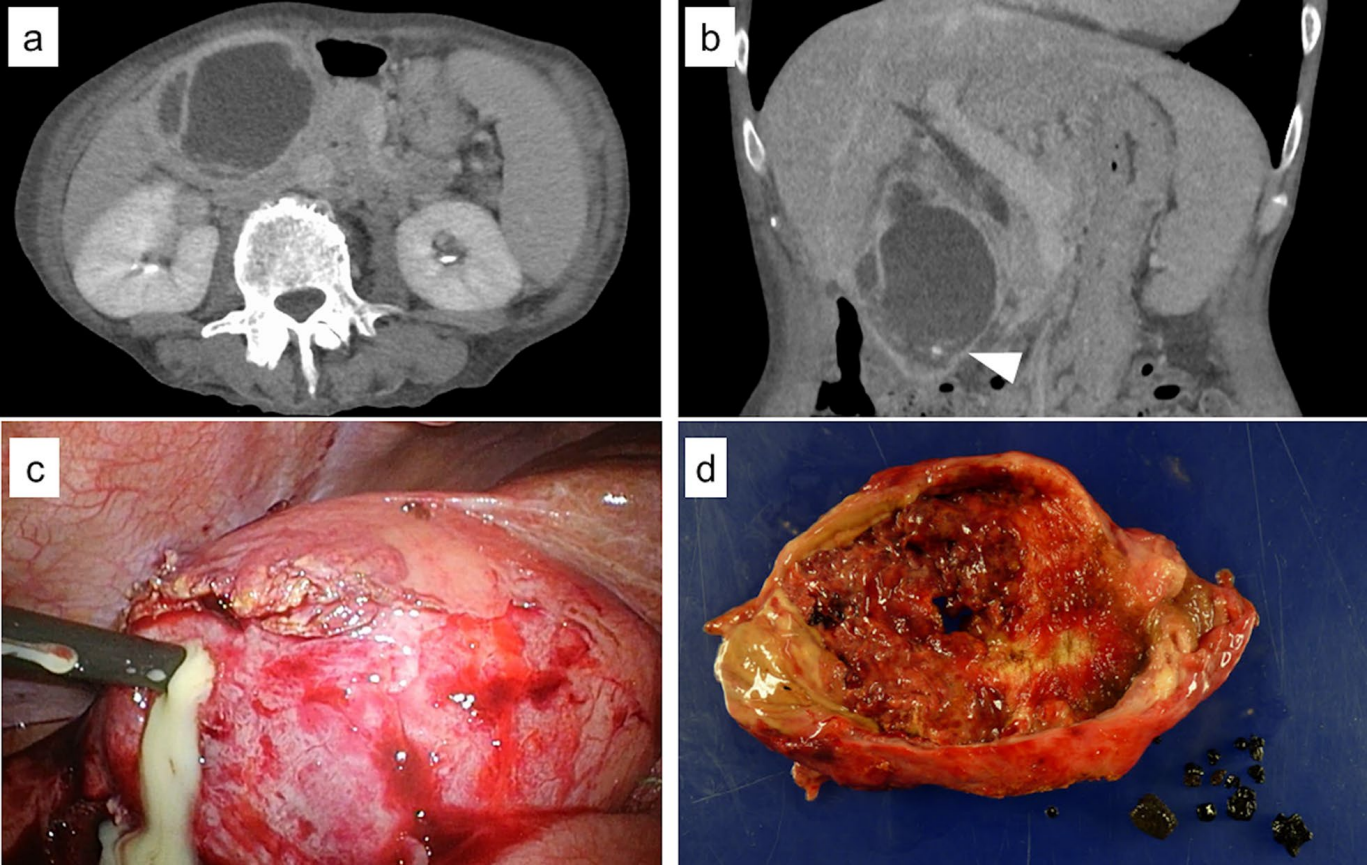
CT scan and gallbladder of initial surgery. Abdominal CT performed at the time of diagnosis of cholecystitis shows marked gallbladder swelling and intramural abscess, with gallstones (arrowhead): **a** axial and **b** coronal views. **c** Laparoscopic cholecystectomy for acute cholecystitis. Iatrogenic perforation of the gallbladder due to the pus drainage. **d** Gallstones and gallbladder with wall thickening and mucosal necrosis

A 66-year-old woman was referred to our hospital because of right upper abdominal pain, right chest pain, and dyspnea. Her past medical history included hypertension, primary biliary cirrhosis, and rheumatoid arthritis (RA), treated with a steroid agent and interleukin-6 (IL-6) inhibitor. In addition, LC was performed for acute gangrenous cholecystitis 11 months earlier (Fig. 1). The operative report revealed iatrogenic gallbladder perforation and stone spillage due to severe regional inflammation. The bacterial culture of the gallbladder bile tested positive for *Escherichia coli*. Tazobactam/piperacillin was administered from the time of surgery to postoperative day 7. Although the inflammatory markers such as white blood cell counts and C-reactive protein were improved, she had persistent fever. Computed tomography (CT) was performed and multiple dropped stones were found. However, abdominal abscess was not found on CT. Upon receiving the culture results, meropenem was administered for the next 10 days. She was discharged from the hospital on postoperative day 21.

**Fig. 2:**
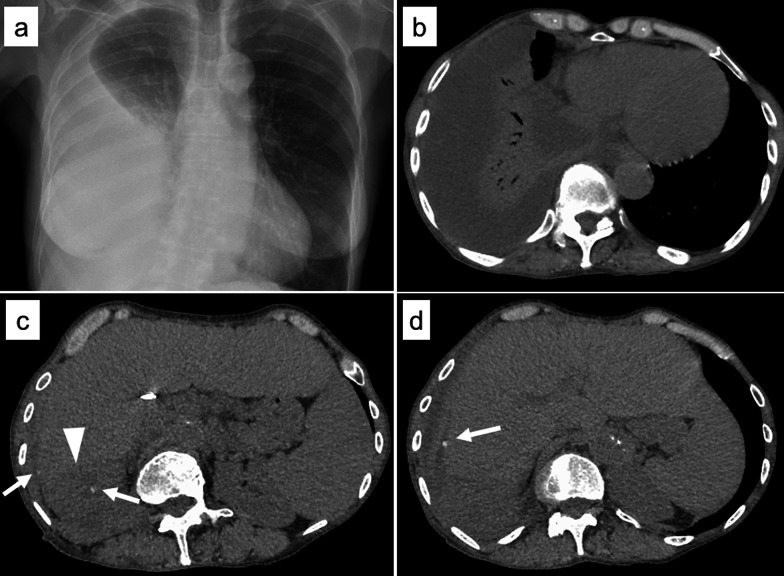
Chest radiography and CT at the time of pleural empyema diagnosis. **a** Chest radiography reveals a large amount of right pleural effusion. **b** Chest CT shows a large amount of right pleural effusion with passive atelectasis. **c**, **d** Abdominal CT shows perihepatic fluid collection (arrowhead) and multiple small radiopaque density masses (arrow)

**Fig. 3:**
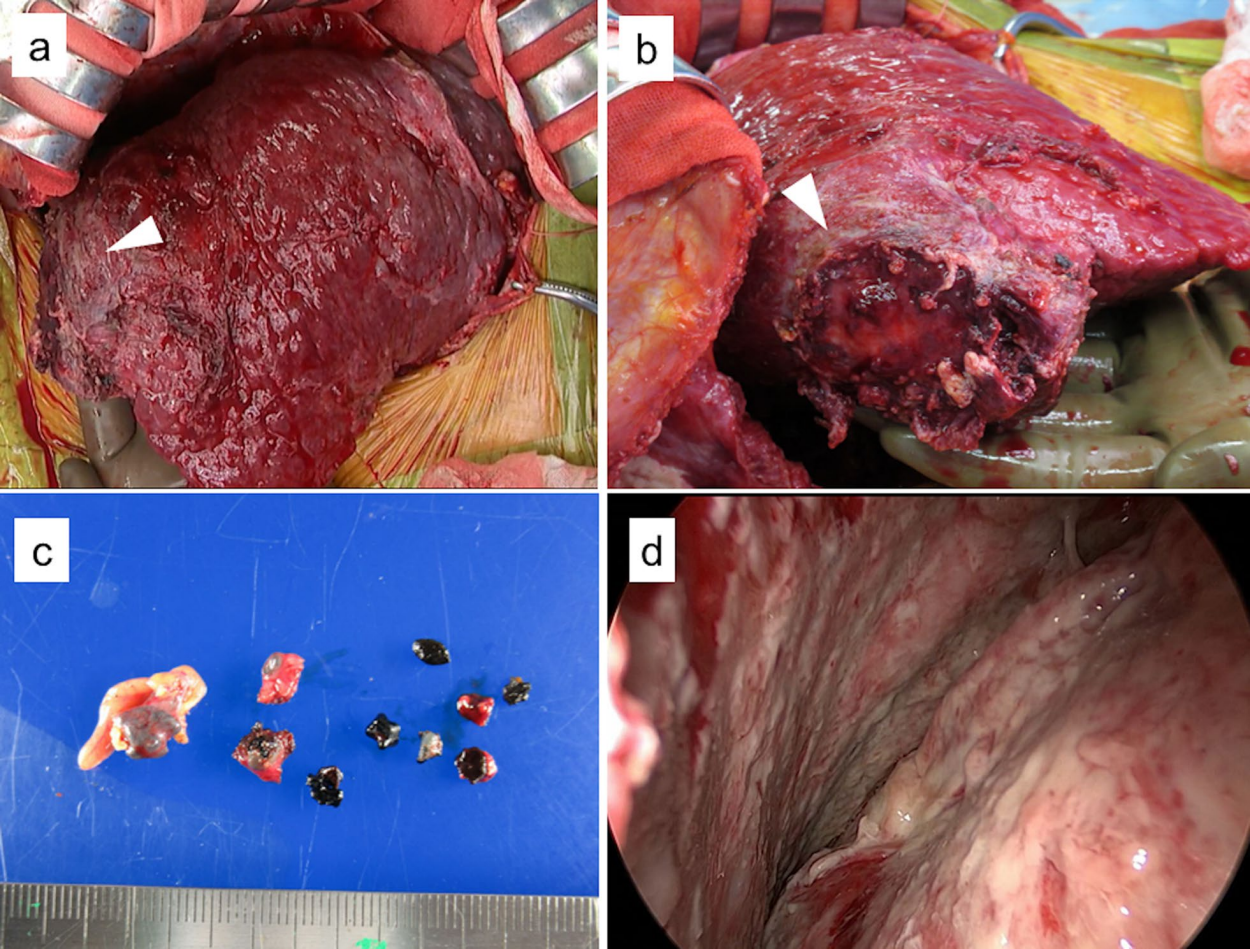
Thoracotomy and laparotomy for drainage. **a**, **b** The abscess in the Morrison's fossa was opened (arrowhead). **c** Retrieved gallstones. **d** Decortication via video-assisted thoracoscopy. No fistula connecting to the abdominal cavity was found

Chest radiography revealed a right pleural effusion (Fig. 2a). Chest CT revealed a right pleural effusion with passive atelectasis (Fig. 2b). Furthermore, abdominal CT showed perihepatic fluid collection and multiple small radiopaque density masses (Fig. 2c). The patient was treated with an antimicrobial agent (meropenem), and ultrasound-guided transthoracic drainage was performed. The bacterial culture of pleural effusion was positive for *Escherichia coli*, which was consistent with the culture result of the gallbladder bile in acute cholecystitis. However, the drainage was incomplete, and the prescribed treatments did not alleviate the inflammatory reaction. Consequently, thoracotomy and laparotomy with gallstone retrieval were performed. In laparotomy, the abscess in the Morrison’s fossa was opened and drained, and dropped stones around the liver were removed (Fig. 3a–c). The thoracic cavity was covered with membranous necrotic tissue, and a multilocular abscess was observed. However, there was no communication between the thoracic cavity and the abdominal cavity and there were no stones in the thoracic cavity (Fig. 3d). The patient’s symptoms, pleural effusion, and perihepatic fluid collection completely resolved after surgery. The patient has remained well without complications after 14 months of follow-up.

## Discussion

This study highlighted two significant findings. First, pleural empyema occurred owing to delayed-onset gallstone formation after LC for acute cholecystitis. Second, complete removal of dropped gallstones was the only curative treatment for pleural empyema.

LC has become the gold standard treatment for cholelithiasis [1–3]. The application of LC for acute cholecystitis has increased owing to improvements in the procedure and reduced postoperative hospital stay. However, difficult LC cases, such as necrotic cholecystitis, have been encountered [8]. Iatrogenic gallbladder perforation reportedly occurred in 10–30% of LC procedures [5]. Moreover, the incidence of dropped gallstones was reportedly 5.4–19%, and 1.1–2.3% of dropped gallstones persisted [6].

The incidence of abdominal abscess formation after LC is reportedly low at 0.08%. However, this increases to 1.46% when dropped gallstones are not retrieved [7]. Therefore, 76.8% of surgeons have followed up their patients with dropped gallstones two years after LC owing to concerns regarding abdominal infections [9]. The development of pleural empyema after LC has rarely been reported, with only 13 previous cases [10–22]. The clinical features of pleural empyema, caused by dropped gallstones after LC, are summarized in Table 1. The median age of the patients was 68 years (range, 53–83 years). The etiologic agents were *Escherichia coli* in five cases, *Klebsiella spp.* in four cases, *Enterococcus spp.* in two cases, and *Salmonella spp.* in one case. The median period from LC to abscess formation was 17 months (range, 1.5–63 months).

**Table 1:** Literature review of pleural empyema caused by dropped gallstones during laparoscopic cholecystectomy

Author		Age (years)/sex	Interval time from cholecystectomy	Treatment for pleural empyema		Complete retrieval of gallstones	Effect of treatment
1	Leslie [10]	58/male	15 months	1st	Thoracotomy	No	Failure
				2nd	Drainage	Yes	Success
2	Barnard [11]	54/female	13 months	1st	Thoracotomy	Yes	Success
3	Willekes [12]	83/female	17 months	1st	Thoracotomy	Yes	Success
4	Chopra [13]	64/female	30 months	1st	Drainage	Yes	Success
5	Preciado [14]	71/male	18 months	1st	Drainage	No	Failure
				2nd	Thoracotomy	Yes	Success
6	Roberts [15]	64/male	17 months	1st	Use of antimicrobials	No	Failure
7	Bergeron [16]	72/female	1.5 months	1st	Drainage	No	Failure
				2nd	Thoracotomy	Yes	Success
8	Cheah [17]	72/male	63 months	1st	Thoracotomy (VATS)	Yes	Success
9	Quail [18]	66/female	60 months	1st	Use of antimicrobials	No	Failure
				2nd	Thoracotomy (VATS)	Yes	Success
10	Gaster [19]	72/male	N/A	1st	Drainage	No	Failure
				2nd	Thoracotomy	Yes	Success
11	Robinson [20]	53/male	60 months	1st	Thoracotomy	Yes	Success
12	McPherson [21]	83/male	N/A	1st	Drainage	No	Failure
13	Tchercansky [22]	69/male	5 months	1st	Drainage	No	Failure
				2nd	Thoracotomy (VATS)	Yes	Success
14	Our case	66/female	11 months	1st	Drainage	No	Failure
				2nd	Thoracotomy	Yes	Success

*VATS* video-assisted thoracic surgery, *N/A* not available

The mechanisms that the abdominal infection causes the pleural empyema were reported as follows [23, 24]: first, a port of laparoscopic surgery or a drainage tube may be placed via the thoracic cavity. Second, the diaphragm has congenital defect, such as the esophageal hiatus and the aortic hiatus. Third, inflammation may have destroyed the diaphragm and formed a fistula. Finally, bacteria may have entered the thoracic cavity through the lymphatic network that is abundant in the diaphragm. In any case, there is negative pressure in the thoracic cavity, and infection in the abdominal cavity may transfer into the thoracic cavity through the diaphragm. However, in our case, cholecystitis was not previously treated with percutaneous transhepatic gallbladder drainage and there was no transthoracic port in laparoscopic cholecystectomy. In the previous reports, the fistula which was caused by abdominal inflammation was identified in the diaphragm [11, 20, 22]. In contrast, the fistula could not be found in the diaphragm by the visual inspection and palpation in our case. Furthermore, there were no stones in the thoracic cavity on CT. However, it is difficult to identify the micro fistula intraoperatively. Therefore, we suspected that there was the micro fistula between the abdominal abscess and thoracic cavity. Furthermore, ultrasound-guided transthoracic drainage and antimicrobial agent were not effective enough because the abscess cavity was multilocular. In these aspects, we needed a thoracotomy with laparotomy for complete drainage.

Complete retrieval of dropped gallstones is necessary for the complete recovery of patients with pleural empyema or an abdominal abscess caused by dropped gallstones. The pathogenic bacteriologic profile of pleural empyema, caused by intra-abdominal infection, suggested an abdominal origin [23]. *Escherichia coli*, *Klebsiella spp.*, and *Enterococcus spp.* have been commonly isolated in acute cholecystitis [25]. In our case, the bacteriological profile of pleural empyema was similar to that of the bile at the time of acute cholecystitis. However, the pleural empyema and abdominal abscess were not completely resolved despite abscess drainage and administration of the appropriate antimicrobials, as shown in Table 1. However, the pleural empyema and abdominal abscess were treated entirely with complete retrieval of the dropped gallstones. Therefore, dropped gallstones should always be retrieved. Furthermore, abscess formation, caused by dropped gallstones, requires a long time to develop. In these cases, a physician aside from the surgeon can detect the development of pleural empyema or an abdominal abscess. Therefore, all physicians should be informed that the treatment of an abscess, due to dropped gallstones, requires prompt and complete retrieval of the dropped gallstones.

In our case, IL-6 inhibitor is considered one of causes diagnosed after reaching pleural empyema. She started IL-6 inhibitor 2 months before the onset of empyema because her rheumatism worsened. While IL-6 inhibitors are highly effective biological drugs for RA, their anti-inflammatory effects masked the signs and symptoms of infection [26, 27]. She also had no fever even at the onset of empyema. Thus, exacerbation of the patient’s condition may have been avoided by recognizing the dropped stones as a source of infection and IL-6 inhibitors as a risk factor for severe infection.

## Conclusion

We report a case of pleural empyema, caused by dropped gallstones after LC for acute cholecystitis. Physicians should keep in mind that dropped gallstones during LC can cause pleural empyema, even after several years. Complete retrieval of the dropped gallstones is necessary to cure pleural empyema and abdominal abscess.

## Data Availability

Data sharing is not applicable as no datasets were generated analyzed during the current study.

## Abbreviations

LC: Laparoscopic cholecystectomy
RA: Rheumatoid arthritis
IL-6: Interleukin-6
